# Supplementary material for: Carboplatin and Etoposide for the Treatment of Metastatic Prostate Cancer with or without Neuroendocrine Features: A French Single-Center Experience
Source: Cancers (Basel). 2024 Jan 9;16(2):280. doi: 10.3390/cancers16020280 (PMC10813788; doi:10.3390/cancers16020280)
Supplement: Supplementary file 1 [file cancers-16-00280-s001.zip › Supplemental_Figure_1.pdf]

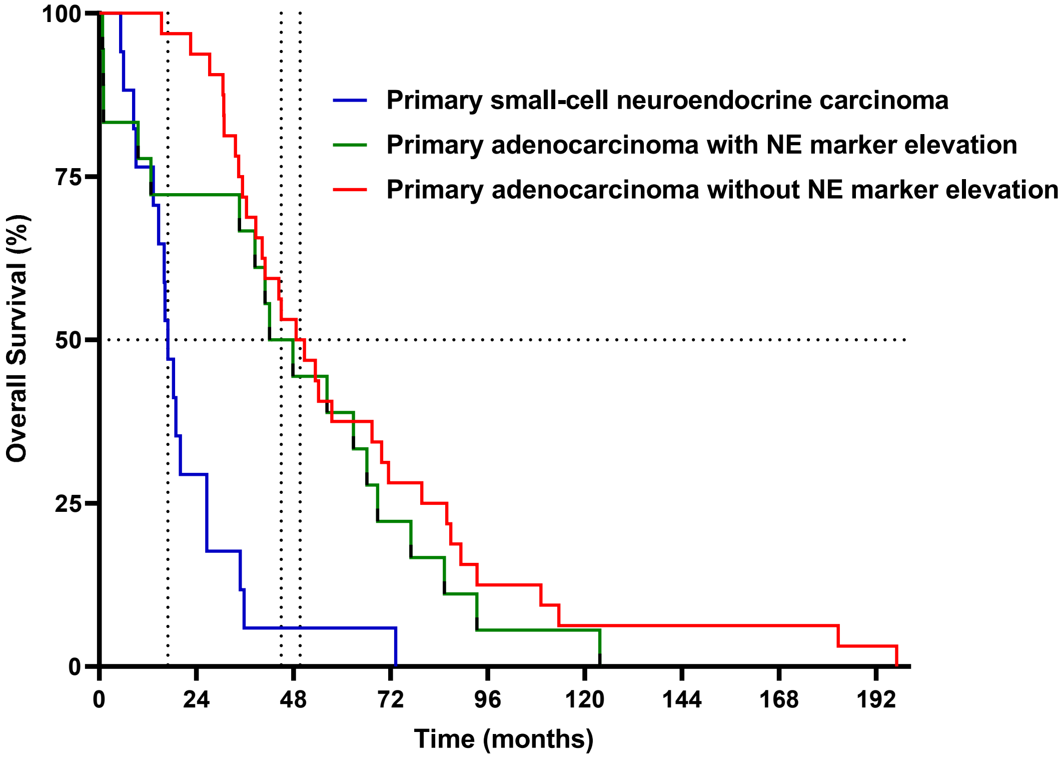

Supplemental Figure 1. Kaplan-Meier estimates of Overall Survival (OS) since diagnosis of metastatic disease

Dashed lines represent median survival. The "primary adenocarcinoma without NE marker elevation" (n=32) and "primary adenocarcinoma with NE marker elevation" (n=18) groups did not differ significantly (p=0.34), but the "primary small cell neuroendocrine carcinoma" (n=17) differed statistically significantly from the other two groups (p<0.01 for both comparisons). Groups were compared using the Log-rank test.

Abbreviations: NE, neuroendocrine
