# Supplementary material for: Carboplatin and Etoposide for the Treatment of Metastatic Prostate Cancer with or without Neuroendocrine Features: A French Single-Center Experience
Source: Cancers (Basel). 2024 Jan 9;16(2):280. doi: 10.3390/cancers16020280 (PMC10813788; doi:10.3390/cancers16020280)
Supplement: Supplementary file 1 [file cancers-16-00280-s001.zip › Supplemental_Table_S1.pdf]

Patients	Previous HNG lines	Other previous systemic therapies
**Primary adenocarcinoma with no elevation of NE markers**		
1	Abiraterone acetate, Enzalutamide	Docetaxel, Cabazitaxel
2	Abiraterone acetate, Enzalutamide	Docetaxel, Cabazitaxel
3	Abiraterone acetate, Enzalutamide	Docetaxel, Cabazitaxel
4	Abiraterone acetate, Enzalutamide	Docetaxel, Cabazitaxel, **Navelbine + Estramustine**
5	Enzalutamide	Docetaxel, Cabazitaxel,
6	Abiraterone acetate, Enzalutamide	Docetaxel, Cabazitaxel
7	Abiraterone acetate	NEXUS vaccine (NEXUS trial), Docetaxel, Docetaxel rechallenge, Navelbine + Estramustine, **Cyclophosphamide**
8	Abiraterone acetate, Enzalutamide	Docetaxel, Cabazitaxel
9	Abiraterone acetate, Enzalutamide	Docetaxel, Cabazitaxel, Radium-223 chloride, Docetaxel rechallenge
10	Abiraterone acetate, Enzalutamide	Docetaxel, Cabazitaxel
11	Abiraterone acetate, Enzalutamide	Docetaxel, Cabazitaxel, Docetaxel rechallenge
12	Enzalutamide	Docetaxel, Cabazitaxel,
13	Enzalutamide	Docetaxel, Cabazitaxel, Docetaxel rechallenge
14	Abiraterone acetate, Enzalutamide	Docetaxel, Cabazitaxel, Olaparib
15	Abiraterone acetate, Enzalutamide	Docetaxel, Cabazitaxel
16	Abiraterone acetate, Enzalutamide	Docetaxel, Cabazitaxel, Docetaxel rechallenge, Cabazitaxel rechallenge
17	Abiraterone acetate, Enzalutamide	Docetaxel, Cabazitaxel, Radium-223 chloride
18	Abiraterone acetate, Enzalutamide	Docetaxel, Cabazitaxel
19	Abiraterone acetate, Enzalutamide	Docetaxel, Cabazitaxel, **Navelbine + Estramustine, Mitoxantrone, Cyproterone acetate**
20	Abiraterone acetate, Enzalutamide	Docetaxel, Cabazitaxel
21	Abiraterone acetate, Enzalutamide	Docetaxel, Cabazitaxel
22	Abiraterone acetate, Enzalutamide	Docetaxel, Cabazitaxel
23		Docetaxel
24	Abiraterone acetate, Enzalutamide	Docetaxel, Cabazitaxel
25	Abiraterone acetate, Enzalutamide	Docetaxel, Cabazitaxel
26	Abiraterone acetate	Docetaxel, Cabazitaxel
27	Enzalutamide	Docetaxel, Cabazitaxel
28	Enzalutamide	Docetaxel, Cabazitaxel, Docetaxel rechallenge
29	Enzalutamide	Docetaxel, Cabazitaxel
30	Abiraterone acetate, Enzalutamide	Docetaxel, Cabazitaxel
31	Abiraterone acetate, Enzalutamide	Docetaxel, Cabazitaxel, Mitoxantrone, Cabazitaxel rechallenge, Durvalumab + Tremelimumab + metronomic Vinorelbin (MOVIE trial)
32	Abiraterone acetate, Enzalutamide	Docetaxel, Cabazitaxel
33		Docetaxel
**Primary adenocarcinoma with elevation of NE markers**		
1		Docetaxel, Navelbine + Estramustine, Docetaxel rechallenge
2		Docetaxel
3	Abiraterone acetate	Docetaxel, Docetaxel rechallenge
4	Abiraterone acetate, Enzalutamide	Docetaxel, Cabazitaxel
5		Docetaxel
6	Abiraterone acetate, Enzalutamide	Docetaxel, Cabazitaxel
7		Docetaxel
8		Docetaxel

9	Abiraterone acetate, Enzalutamide	Docetaxel, Cabazitaxel, Docetaxel rechallenge
10	Abiraterone acetate, Enzalutamide	Docetaxel, Tasquinimod (IPSEN TASQ 002 trial), Docetaxel rechallenge, Cabazitaxel, Radium-223 chloride, Cabazitaxel rechallenge
11	/	
12	/	
13	Abiraterone acetate, Enzalutamide	Docetaxel, Cabazitaxel, Lutetium-PSMA (VISION trial), Durvalumab + Tremelimumab + metronomic Vinorelbine (MOVIE trial)
14	Abiraterone acetate, Enzalutamide	Docetaxel
15	Abiraterone acetate	Docetaxel, Radium-223 chloride, Cabazitaxel
16	Abiraterone acetate	Docetaxel, Cabazitaxel, Docetaxel rechallenge
17		Docetaxel, Navelbine
18	Enzalutamide	Docetaxel, Cabazitaxel

Supplemental Table 1. Individual data concerning previous treatmens received by each patients in the « primary adenocarcinoma » group.
