# Supplementary material for: Carboplatin and Etoposide for the Treatment of Metastatic Prostate Cancer with or without Neuroendocrine Features: A French Single-Center Experience
Source: Cancers (Basel). 2024 Jan 9;16(2):280. doi: 10.3390/cancers16020280 (PMC10813788; doi:10.3390/cancers16020280)
Supplement: Supplementary file 1 [file cancers-16-00280-s001.zip › Supplemental_Table_S2.pdf]

Supplemental Table 2 : Last previous treatment before carboplatin etoposide in the 49 patients included in the individual PFS ratio analysis

	Overall population	primary adenocarcinoma with NE marker elevation	primary adenocarcinoma without NE marker elevation
**N**	49	16	33
**NHA (%)**	11 (22.4)	2 (12.5)	9 (27.3)
**Taxanes (%)**	28 (57.2)	10 (62.5)	18 (54.5)
**Others (%)**	10 (20.4)	4 (25.0)	6 (18.2)

Other treatments were: Radium-223 (3), Durvalumab + Tremelimumab (2), Vinorelbine + Estramustine phosphate (1), Vinorelbine (1), Cyclophosphamide (1), Cyproterone Acetate (1), Olaparib (1)
Abbreviations: NHA, next-generation hormonal agent. NE, neuroendocrine
